# Probiotic and cultural characteristic of strain *Lactobacillus gasseri* 4/13 of human origin

**DOI:** 10.1080/13102818.2014.974303

**Published:** 2014-10-31

**Authors:** Kalinka Baltova, Zhechko Dimitrov

**Affiliations:** ^a^ R & D Center, LB-Bulgaricum PLC, Sofia, Bulgaria

**Keywords:** *Lactobacillus gasseri*, probiotic properties, cultural characteristic, yoghurt

## Abstract

*Lactobacillus gasseri* within the *Lactobacillus acidophilus* group is a major species in the human microflora. The potential probiotic properties of a *L. gasseri* strain of human origin were evaluated. Out of 17 studied *L. gasseri* strains, *L. gasseri* 4/13 showed the highest immunomodulatory effect (induction of interferon gamma measured by sandwich enzyme-linked immunosorbent assay) in Balb/c mouse splenocytes *in vitro* and the highest rate of adhesion to Caco-2 human epithelial cells. The strain also reduced the concentration of cholesterol in the growth medium by 65% as compared with the initial concentration (measured spectrophotometrically). These probiotic properties indicate that *L. gasseri* 4/13 could prove an attractive concentrated adjunct monoculture in the production of new functional foods. To obtain a freeze-dried bacterial concentrate from *L. gasseri* 4/13, the influence of different culture media, temperatures and pH values on the accumulation of cell biomass was studied. Yoghurt samples were produced using a classical fermentation technology. Freeze-dried concentrated monoculture of *L. gasseri* 4/13 with over 1 × 10^10^ CFU/g viable cells was added as an adjunct culture together with a starter. The viable *L. gasseri* 4/13 cells remained above the critical value of 10^6^ CFU/g during storage at 5 °C for the entire 20-day period. Organoleptic tests did not reveal any adverse change in the product taste and aroma of yoghurt samples at the 20th day. In conclusion, *L. gasseri* 4/13 was selected as having suitable probiotic and cultural characteristics for production of fermented milk products with high nutritional and biological value.

## Introduction

In recent years, there has been growing public awareness about the relationship between food and health. The desire of consumers to use natural methods to maintain their health and to prevent various diseases increases the production and consumption of probiotic foods.[1] The consumption of fermented milk products containing live micro-organisms with useful properties has beneficial effects on the body by improving the balance of intestinal microflora.[2] Probiotics are defined as cultures of live micro-organisms which have a beneficial effect on human health during passage through the gastrointestinal tract.[3] To be considered probiotic, bacteria should cover a number of criteria: to be safe, to be stable during the technological process and exert minimal organoleptic impact on the probiotic foods, to resist while passing through the digestive tract, to adhere to intestinal epithelial cells and to have a healthy effect. These effects include the prevention or amelioration of diarrhoea and prevention of cancer and anti-metabolic syndrome actions.[2,4,5] *Lactobacillus gasseri* within the genus of *Lactobacillus acidophilus* is a major species of the human flora and has been widely utilized as a probiotic.[6,7] Many healthy effects of *L. gasseri* have been reported: inhibitory activity against some pathogenic and food-spoilage species, lowering of serum cholesterol concentrations and immune-modulatory effects.

A number of studies show the health benefits of fermented milks containing *L. gasseri* strains.[8–10] Azuma et al.[11] studied the effect of milk fermented with *L. gasseri* on the faecal microbiota of healthy people and showed that the intake of 195 g per day of this fermented milk for nine days has a positive influence on the growth of the living *Bifidobacteria* in the faecal flora and decreases the *Clostridium perfringens* counts.

The overall aim of the present study was to determine the culture conditions for the production of a concentrated freeze-dried culture of a selected probiotic *L. gasseri* strain 4/13 and to determinate the level of *L. gasseri* strain 4/13 added as adjunct culture in the yoghurt during storage.

## Materials and methods

### Probiotic bacteria and starter cultures

For the purpose of this study, the strain *L. gasseri* 4/13 of human origin and three commercial freeze-dried Direct Vat Set (DVS) yoghurt starter cultures LBB 41-8, LBB 5-54V and LBB 435 from the LBB culture collection (LB Bulgaricum PLC, Sofia, Bulgaria) were used. *L. gasseri* strain 4/13 was chosen among the 17 studied *L. gasseri* strains as the best adhering strain towards the human epithelium. All the 17 *L. gasseri* strains were isolated from the faeces of healthy volunteers (data not shown). *L. gasseri* strain 4/13 was maintained in MRS (de Man–Rogosa–Sharpe) broth (16 h, 37 °C), harvested by centrifugation (8000*g*, 20 min, 4 °C) and then stored at −196 °C in liquid nitrogen. A cryoprotector was added to the cells. Before use, *L. gasseri* 4/13 was activated by threefold to fivefold reinoculation in MRS broth and cultivated at 37 °C for 16 h.

### Quantitative assessment of the adhesion

The intestinal cell culture Caco-2 was used in the adhesion assay. This human colon adenocarcinoma cell line was obtained from the American Type Culture Collection. The cells were cultured in Dulbecco's modified Eagle's minimal essential medium (DMEM; GIBCO-BRL), containing 25 mmol/L of glucose, 20% (vol/vol) heated inactivated fetal calf serum (GIBCO-BRL) and 1% nonessential amino acids (GIBCO-BRL). The cells were grown at 37 °C in 5% CO_2_. At approximately 95% confluence, the monolayers were detached by incubating with a 0.25% trypsin solution (Gibco) for 5 min at 37 °C. For the adhesion assay, monolayers of Caco-2 cells were prepared in two-chamber slides (Lab-Tek chamber slide; Nunc Inc.) by inoculating 2.8 × 10^5^ viable cells into 2 mL of culture medium. The medium was replaced every two days. Fifteen-day post-confluent Caco-2 monolayers were washed three times before the adhesion assay with 1 mL of sterile phosphate-buffered saline (PBS). One millilitre of the test bacteria at concentrations between 1 × 10^5^ and 4 × 10^8^ CFU/mL were added to 1 mL of complete Caco-2 medium. This suspension (2 mL) was added to each chamber of the two-chamber slide and incubated at 37 °C, in an atmosphere of 5% CO_2_ and 95% air, with gentle shaking. After incubation for 60 min, the monolayers were washed five times with sterile PBS (pH 7.2), fixed with methanol, Gram stained and examined microscopically. Each adherence assay was conducted in triplicate and the number of bacteria adherent on about 1000 Caco-2 cells was counted in 20 randomly selected microscopic fields. To simulate the physiological pH condition of the gastrointestinal tract, all experiments were done at pH 7. The level of adhesion on Caco-2 human epithelium cells was expressed as the number of attached bacterial cells on one average eukaryotic cell.

### Evaluation of cholesterol-reducing properties of *L. gasseri* strain 4/13

The direct absorption of cholesterol was measured according to Gilliland et al.[12] The concentration of cholesterol in the growth medium was measured spectrophotometrically at 450 nm.

### Evaluation of immunomodulatory effect of *L. gasseri* strain 4/13

For this purpose, *in vitro* tests using spleen cells from the Balb/c mouse line were used. The concentration of interferon gamma produced by splenocytes was measured by sandwich enzyme-linked immunosorbent assay (ELISA) as previously described.[13]

### Production of concentrated culture of *L. gasseri* strain 4/13

Fermentation with *L. gasseri* 4/13 was performed on three media. Medium 1 was standard MRS broth (Oxoid). Media 2 and 3 were milk based and contained lactose, yeast extract and peptone casein. Media 2 and 3 differed in the content of skim milk powder, which was 6% and 8%, respectively.

The effect of active acidity (pH) on the cultivation of *L. gasseri* 4/13 was tested in Medium 1. The values of pH at which the strain was cultivated were 5.7, 5.9 and 6.2.

To test the influence of different culture media and pH on the cultivation of the probiotic, *L. gasseri* 4/13 was cultured at laboratory scale in a New Brunswick Scientific (USA) Bioflo 2000 bioreactor. Five per cent of *L. gasseri* 4/13 mother starter was inoculated into the media and the fermentation was carried out with pH control by automatic feeding of neutralizing solution (20% NaOH, w/v). At the end of the fermentation, the cell mass was separated by centrifugation (10,000*g* for 20 min, at 4 °C). Cryoprotectors (10%, v/v) were added to the biomass fraction and lyophilization was performed in a freeze-drying machine (Labconco).

### Production of fermented milks and analysis of final product

The technology for the production of Bulgarian yoghurt was used as a basis for the experiments. Whole cow's milk homogenized and pasteurized at 95 °C with a delay of 10 min was cooled to inoculation temperature of 43 °C. Then, DVS freeze-dried yoghurt starter cultures LBB.BY41-8, LBB.BY5-54V or LBB.BY435 were added by stirring. Along with traditional starter cultures, *Lactobacillus delbrueckii* subsp*. bulgaricus* and *S. thermophilus* the *L. gasseri* 4/13 were added as freeze-dried concentrated adjunct culture in a 0.03%–0.05% (w/v) volume. The fermentation process continued at 43 °C for about 4.5–5 h to pH 4.7 ± 0.2. Then, milk samples were refrigerated immediately and stored at 5 °C for 21 days. The spread plate technique was used to determine viable cell counts (CFU/mL). Tenfold serial dilutions were prepared and plated on MRS or M17 agar to score *L. delbrueckii* subsp*. bulgaricus* and *S. thermophilus* cells, respectively. Plates were incubated for 72 h at 37 °C. All platings were performed in duplicate. To score *L. gasseri*, the standard ISO 20128/2006 was used.

### Physicochemical and sensory analyses

The pH of yoghurt samples was measured at 1, 7, 14 and 21 days of storage at 5 °C by using a METTLER TOLEDO MP 220 pH-Metre. The hardness of the coagulum was determined with a Curd Meter Max ME-305/HI-80 equipped with a plotter. A 100 g weight distributed on a 20-mm disk was gradually lowered to the surface of the product. The unit recorded the weight applied at the moment of breaking of the coagulum. Whey separation was measured by centrifugation of 50*g* product at 3000 r/min for 10 min and measuring the weight of the supernatant. The syneresis was calculated by the formula:


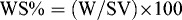


where WS is the value of syneresis, *W* is the weight of the supernatant (g) and SV is the weight of the initial sample (g).

## Results and discussion

In line with the ever-increasing consumer demand for healthy and functional foods worldwide, we studied 17 probiotic *L. gasseri* strains of human origin for their potential use as probiotic adjunct cultures in the production of yoghurt.

### Probiotic properties of the strains

The adhesive properties of *L. gasseri* strains were evaluated using Caco-2 human epithelium cells. The result from the assay of adhesion of 17 *L. gasseri* strains is shown in Table 1. The average number of attached bacteria on one average eukaryotic cell ranged between 1 and 17. The average number of *L. gasseri* 4/13 cells adhered onto one Caco-2 cell was 17 and this strain was chosen as the best adhering strain to human epithelium.

**Table 1.** Average number of adhered bacteria per eukaryotic Caco-2 cell in the adhesion experiment.

*L*. *gasseri* strain	Adhered bacteria	*L*. *gasseri* strain	Adhered bacteria
4/13	17	1/14	4
7/12	16	4/7	4
11/14	14	4/9	3
8/1	11	3/14	3
3/15	9	2/10	3
5/10	6	2/20	2
4/15	6	3/1	2
1/11	4	5/15	1
5/12	4		

The results from the assessment of the cholesterol-reducing activity of the 17 *L. gasseri* strains are given in Table 2. Strain *L. gasseri* 4/13 demonstrated the second best cholesterol-reducing activity after strain *L. gasseri* 7/12. The rate of cholesterol reduction achieved by strain *L. gasseri* 4/13 was 65% as compared to the initial concentration of cholesterol in the growth medium.

**Table 2.** Rate of cholesterol reduction as compared with the initial concentration of cholesterol in the growth medium.

*L*. *gasseri* strain	Percentage of reduction	*L*. *gasseri* strain	Percentage of reduction
7/12	72.1	1/11	17.0
4/13	65.0	5/10	17.0
11/14	61.2	3/14	14.2
8/1	53.7	2/20	13.5
4/15	37.4	4/9	11.2
5/12	32.0	2/10	8.7
1/14	27.3	5/15	7.5
3/15	19.8	3/1	4.6
4/7	19.5		

The cytotoxic immunomodulatory effect of the strains was studied by the means of an *in vitro* test using mouse splenocytes obtained from the Balb/c mouse line. The induction of interferon gamma was measured as one of the main cytokines indicative of stimulation of the cytotoxic immune response of the host organism. The results from the sandwich ELISA test (Table 3) showed that strain *L. gasseri* 4/13 induced the highest increase in the concentration of interferon gamma (5.7 pg/mL) produced by splenocytes, as compared with the other studied *L. gasseri* strains.

**Table 3.** Concentration of interferon gamma (IF-γ) produced by splenocytes induced by the studied *L. gasseri* strains.

*L*. *gasseri* strain	IF-γ (pg/mL)	*L*. *gasseri* strain	IF-γ (pg/mL)
4/13	5.7	4/7	0.5
7/12	4.6	2/10	0.2
8/1	3.8	3/14	nd
11/14	3.2	2/20	nd
4/15	2.7	1/14	nd
1/11	2.7	5/15	nd
5/12	1.2	4/9	nd
3/15	1.0	3/1	nd
5/10	0.7		

Note: nd – not detected.

Based on its good adhesive properties, satisfactory cholesterol-reducing activity and immunomodulatory effect, *L. gasseri* 4/13 was selected as the strain with best probiotic properties and was used in the second stage of our experiments.

### Effect of different culture media on the accumulation of cell mass

**Figure 1.**
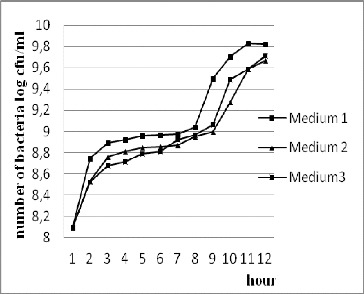
Influence of the composition of culture media on the growth of strain *L. gasseri* 4/13 during cultivation in fermentor system at 37 °C, pH 6.2.

In the process of cultivation in a fermentor system with constant parameters (37 °C, pH 6.2), strain *L. gasseri* 4/13 produced cell mass of about 3.15 × 10^9^ CFU/mL to 6.65 × 10^9^ CFU/mL at 11 h of fermentation (Figure 1). The stationary growth phase of *L. gasseri* 4/13 in all three media occurred at 11–12 h of the fermentation process. In all three media during the course of fermentation, the cell mass reached over 3.15 × 10^9^ CFU/mL at 10–11 h of cultivation. Since the highest biomass was accumulated in Medium 1, this medium was used in the subsequent experiments.

### Effect of pH on the accumulation of cell mass

**Figure 2.**
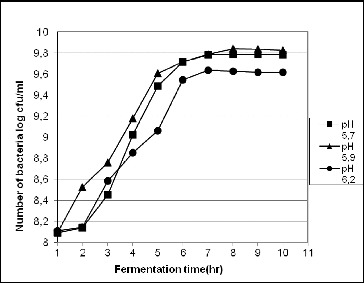
Influence of the acidity (pH) on the growth of strain *L. gasseri* 4/13 during cultivation in the fermentor system at 37 °C.

The experiment was carried out during the fermentation of strain *L. gasseri* 4/13 in Medium 1. The results presented in Figure 2 show that at 8–9 h, the accumulated cell mass at pH 5.9 5.7 and 6.2 was 6.91 × 10^9^, 6.15 × 10^9^ and 4.25×10^9^ CFU/mL, respectively.

### Preparation of fermented milks

To perform the experiments for the preparation of fermented milks, a freeze-dried culture of *L. gasseri* 4/13 with a number of viable cells of 4.49 × 10^11^ CFU/g was used.

The analysis of the values of the technological, organoleptic and microbiological parameters in dynamics showed that the experimental fermented milks had similar parameters. All three starters had a low post-acidification activity. The freshly produced yoghurt samples after 24-h refrigerated storage had a pH value of 4.70–4.69, which decreased gradually, but after 21 days it was still in the range of pH 4.38–4.32. Whey separation remained low for the whole period of observation. The hardness of the coagulum was similar for yoghurts produced with three starters and slightly increased during storage (Table 4). Organoleptic tests did not reveal any adverse change in the taste and aroma of yoghurt samples at day 21 (data not shown).

**Table 4.** Technological parameters of probiotic yoghurt fortified with *L. gasseri* 4/13 during storage (21 days, 5 °C).

Product	Storage (days)	pH	Hardness (g)	Whey separation (%)
Yoghurt with starter BY 41-8	1	4.69	33	18.90
	7	4.47	32	18.50
	14	4.36	32	19.21
	21	4.32	34	19.12
				
Yoghurt with starter BY 5-54V	1	4.69	34	19.01
	7	4.48	33	19.42
	14	4.44	33	19.56
	21	4.38	32	19.81
				
Yoghurt with starter BY 435	1	4.70	32	19.81
	7	4.49	32	19.42
	14	4.35	33	18.16
	21	4.33	33	18.00

**Figure 3.**
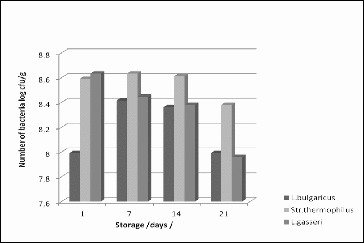
*L. gasseri* bacterial counts during the storage of yoghurt produced with the BY 5-54V starter.

The viable cells of *L. gasseri* 4/13 decreased from 4.3 × 10^8^ CFU/mL at the first day to 9.1 × 10^7^ CFU/mL at day 21. They remained above the critical threshold of 10^6^ CFU/mL over the entire period of 21 days during storage (Figure 3).

Taken together, our results showed that *L. gasseri* 4/13 proved to have suitable probiotic and cultural characteristics for the production of fermented milk products with high nutritional and biological value: excellent level of adhesion to Caco-2 cells, good ability to reduce the concentration of cholesterol, ability to induce the production of interferon gamma and high concentration of live cells in the product during storage. According to many authors, these properties are of high importance for the selection of probiotic strains.[14,15]

## Conclusions

Strain *L. gasseri* 4/13 was shown to combine high level of adhesion to human epithelium with good ability to reduce the cholesterol concentration and a beneficial potential to stimulate the immune system. The strain was successfully applied as an adjunct culture to yoghurt starters, demonstrating excellent technological properties. Yoghurt products enriched with *L. gasseri* 4/13 had a well-accepted taste and high concentration of live *L. gasseri* 4/13 cells.
